# Habituation of phase-locked local field potentials and gamma-band oscillations recorded from the human insula

**DOI:** 10.1038/s41598-018-26604-0

**Published:** 2018-05-29

**Authors:** Giulia Liberati, Maxime Algoet, Anne Klöcker, Susana Ferrao Santos, Jose Geraldo Ribeiro-Vaz, Christian Raftopoulos, André Mouraux

**Affiliations:** 1 Institute of Neuroscience, Université catholique de Louvain, 1200 Brussels, Belgium; 2 Department of Neurology, Saint-Luc University Hospital, 1200 Brussels, Belgium; 3 Department of Neurosurgery, Saint-Luc University Hospital, 1200 Brussels, Belgium

## Abstract

Salient nociceptive and non-nociceptive stimuli elicit low-frequency local field potentials (LFPs) in the human insula. Nociceptive stimuli also elicit insular gamma-band oscillations (GBOs), possibly preferential for thermonociception, which have been suggested to reflect the intensity of perceived pain. To shed light on the functional significance of these two responses, we investigated whether they would be modulated by stimulation intensity and temporal expectation – two factors contributing to stimulus saliency. Insular activity was recorded from 8 depth electrodes (41 contacts) implanted in the left insula of 6 patients investigated for epilepsy. Thermonociceptive, vibrotactile, and auditory stimuli were delivered using two intensities. To investigate the effects of temporal expectation, the stimuli were delivered in trains of three identical stimuli (S1-S2-S3) separated by a constant 1-s interval. Stimulation intensity affected intensity of perception, the magnitude of low-frequency LFPs, and the magnitude of nociceptive GBOs. Stimulus repetition did not affect perception. In contrast, both low-frequency LFPs and nociceptive GBOs showed a marked habituation of the responses to S2 and S3 as compared to S1 and, hence, a dissociation with intensity of perception. Most importantly, although insular nociceptive GBOs appear to be preferential for thermonociception, they cannot be considered as a correlate of perceived pain.

## Introduction

Thermal nociceptive stimuli elicit robust low-frequency phase-locked local field potentials (LFPs) in the human insula^1–4^, which are often assumed to reflect early stages of cortical processing specifically related to nociception and to the perception of pain^2,3^. Contrasting with this assumption, we recently observed that intense but non-nociceptive and non-painful tactile, auditory, and visual stimuli elicit similar low-frequency phase-locked LFPs at the same insular locations^1^. Therefore, both nociceptive and non-nociceptive low-frequency phase-locked LFPs appear to predominantly reflect multimodal neural activity unspecific for pain and nociception^1^. One possibility is that these responses reflect activity involved in detecting, orienting attention towards, and reacting to the occurrence of *salient* sensory events, i.e., stimuli that stand out relative to the sensory background or with respect to preceding stimuli, regardless of their painfulness^5–7^.

In addition to low-frequency phase-locked LFPs reflecting multimodal activity, we recently showed that nociceptive stimuli also elicit an early-latency (150–300 ms) enhancement of gamma-band oscillations (GBOs, >40 Hz) in the human insula, which is not observed in response to non-nociceptive tactile, auditory, and visual stimuli perceived as equally intense and arousing^8^. Hence, differently from multimodal low-frequency phase-locked LFPs recorded at the same locations, stimulus-evoked GBOs recorded from the human insula appear to reflect activity that is *preferential* for thermonociception and/or the processing of spinothalamic input.

The aim of the present study was to shed light on the functional significance of both low-frequency phase-locked LFPs and high-frequency non-phase-locked GBOs recorded from the human insula, by investigating how these responses are affected by the intensity of stimulation, as well as by a manipulation of temporal expectancy, which impacts their saliency. More specifically, we used a paradigm that allows to increase the temporal expectancy of the stimuli by repeating the stimulus at a constant and predictable inter-stimulus interval (ISI).

A number of magnetoencephalography (MEG) and scalp electroencephalography (EEG) studies have shown that stimulus repetition at a constant ISI leads to a strong decrease in the amplitude of phase-locked responses, and in particular of laser-evoked potentials (LEPs) – a phenomenon defined as response *habituation*^5,9–17^. Because this response habituation is not necessarily paralleled by a reduction in the intensity of perception, this can lead to a marked dissociation between the magnitude of these event-related potentials and perception^13^. Differently from these phase-locked responses, GBOs elicited by thermonociceptive stimuli over the human primary somatosensory cortex (SI) have been suggested to not habituate when stimuli are presented at a constant and predictable ISI^18^, indicating that these high-frequency activities might reflect processes more directly related to the perception of pain and/or the strength of the eliciting sensory input.

**Figure 1:**
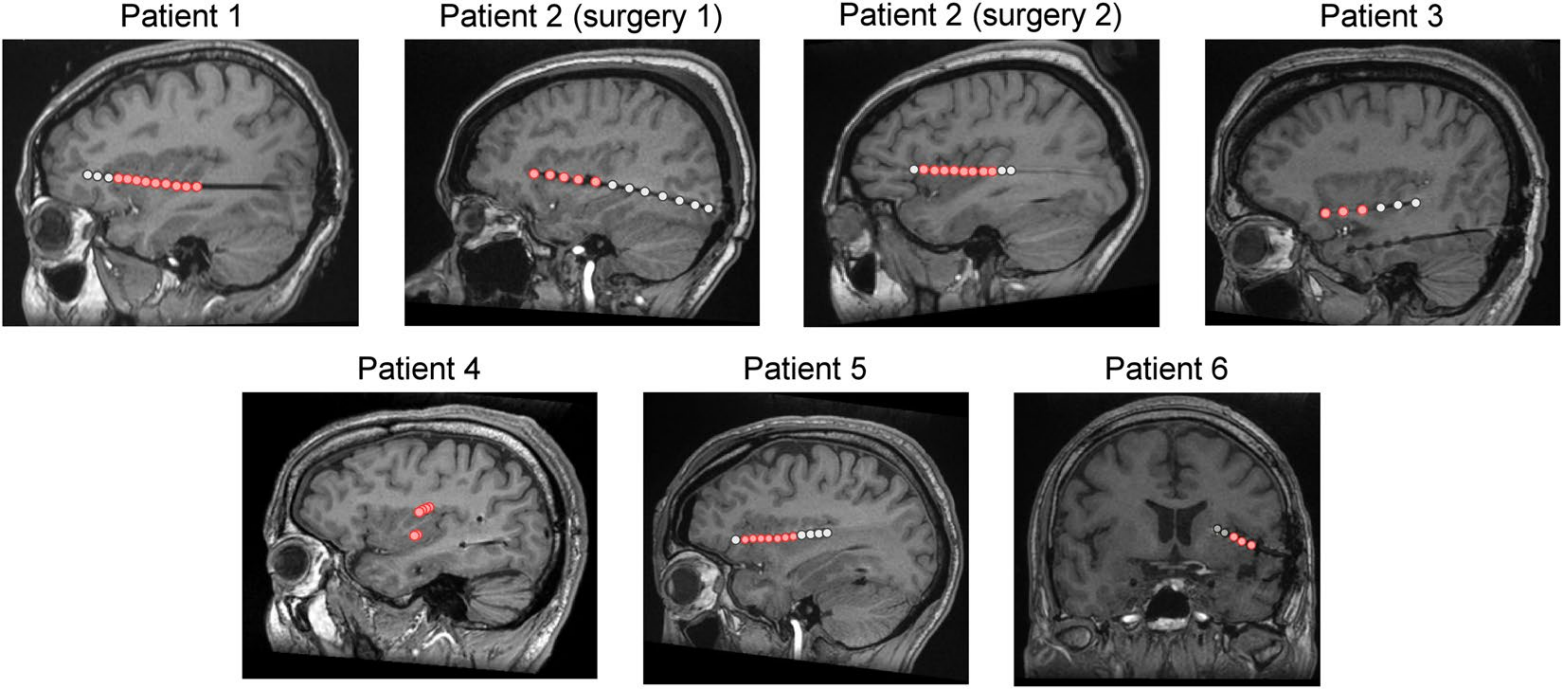
Localization of the 8 depth electrodes implanted in the insula of 6 patients. All electrodes were located in the left insula. A total of 41 insular sites (in red) were investigated. MNI coordinates of the electrode contacts are presented in Table 1. The DICOM images of each patient are available at the OSF online repository at the address www.osf.io/yw4nf.

**Figure 2:**
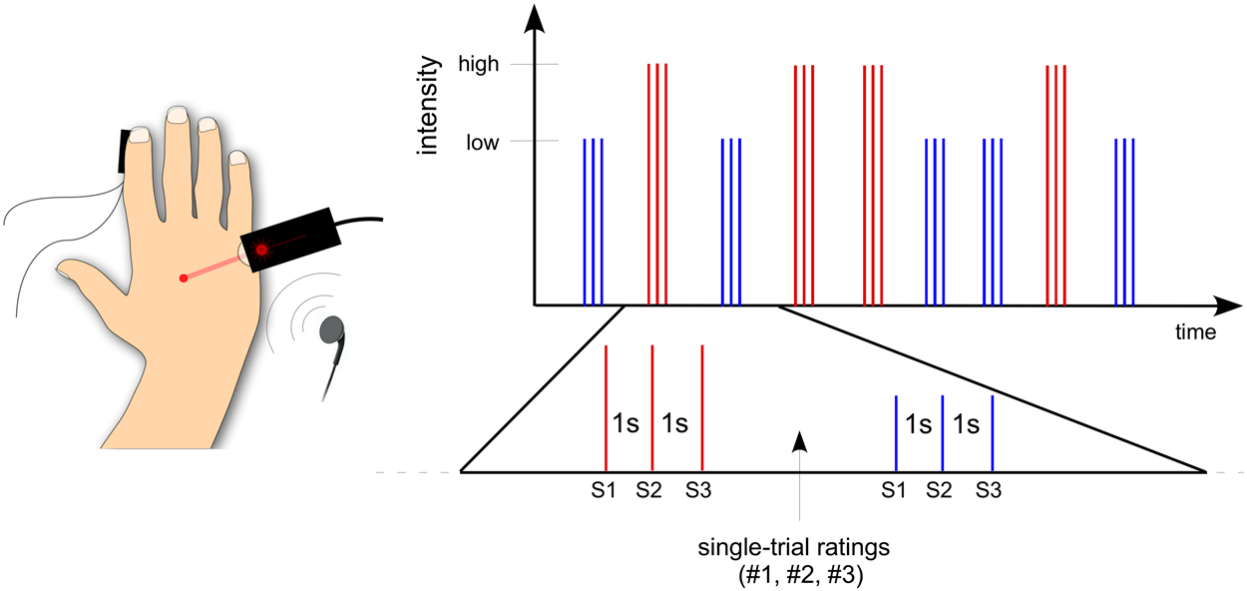
Experimental procedure. Participants received nociceptive, vibrotactile, and auditory stimuli in a randomized block design. To investigate the effect of stimulus expectancy, in each block, trains of three stimuli of identical intensity (S1-S2-S3, “triplets”) were delivered at a constant and predictable inter-stimulus interval of 1 s. Two different intensities, “high” (in red) and “low” (in blue) triplets, were used for each modality, and presented in a randomized order. At the end of each “triplet”, participants rated the intensity of each stimulus on a numerical scale ranging from 0 to 10.

Using 8 depth electrodes implanted in the left anterior and posterior insula of 6 patients (3 females; age: 23–51 years) undergoing a pre-surgical evaluation of focal epilepsy, we recorded intracerebral EEG from a total of 41 insular sites (Fig. 1; see Table 1 for the MNI coordinates of the contacts). Participants received brief thermonociceptive laser stimuli, mechanical vibrotactile stimuli, and auditory stimuli in three separate blocks, in a randomized block design (Fig. 2). In each block, trains of three stimuli of identical intensity (S1-S2-S3, a “triplet”) were delivered at a constant and predictable ISI of 1 s, similarly to a number of previous studies investigating the effect of stimulus repetition on brain responses using scalp EEG^5,13,16–18^. For each modality, two different triplet intensities (“high” and “low”) were used, presented in a randomized order. At the end of each triplet, participants rated the intensity of each of the three stimuli on a numerical scale ranging from 0 to 10, where “0” corresponded to “no perception at all”, and “10” corresponded to “maximum intensity imaginable”. Furthermore, at the end of each block, participants were asked to report whether they had perceived the stimuli as painful.

**Table 1:** Localization of insular electrode contacts.

Subject	Hemisphere	Contact	Description	MNI coordinates
1	Left	1	Anterior insular cortex, adjacent to the opercular portion of the inferior frontal gyrus	−34, 22, −2
		2	Anterior insular cortex, first short gyrus	−35, 16, −2
		3	Anterior insular cortex, transition between the first and second short gyri	−35, 11, −2
		4	Anterior insular cortex, second short gyrus	−35, 5, −2
		5	Posterior insular cortex, subcortical portion of the first long gyrus	−36, −1, −3
		6	Posterior insular cortex, subcortical portion of the first long gyrus	−36, −6, −3
		7	Posterior insular cortex, subcortical portion of the second long gyrus	−37, −12, −3
		8	Posterior insular cortex, subcortical portion of the second long gyrus	−37, −17, −3
		9	Posterior insular cortex, subcortical portion of the circular fold, adjacent to the superior temporal gyrus	−37, −23, −3
2 (first surgery)	Left	1	Anterior insular cortex, first short gyrus, posterior to the circular fold	−30, 25, −1
		2	Anterior insular cortex, subcortical portion of the second short gyrus	−30, 19, −1
		3	Anterior insular cortex, third short gyrus	−30, 17, −1
		4	Posterior insular cortex, first long gyrus	−31, 13, −2
		5	Posterior insular cortex, second long gyrus	−32, 9, −2
3	Left	1	Anterior insular cortex, adjacent to the anterior portion of the circular sulcus	−38, 11, −11
		2	Anterior insular cortex, first short gyrus	−38, 1, −9
		3	Anterior insular cortex, second short gyrus	−39, −9, −6
4a	Left	1	Posterior insular cortex, external capsule	−33, −12, 13
		2	Posterior insular cortex, transition between the long gyrus of the insula and the cortex from the parietal operculum, at the level of the circular gyrus	−37, −12, 15
		3	Posterior insular cortex, transition between the long gyrus of the insula and the cortex from the parietal operculum, at the level of the circular gyrus	−41, −12, 16
		4	Posterior insular cortex / parietal operculum	−49, −12, 16
4b	Left	1	Posterior insular cortex, most posterior long gyrus	−38, −9, −5
		2	Posterior insular cortex, most posterior long gyrus	−42, −9, −5
5	Left	1	Anterior insular cortex, adjacent to the inferior frontal gyrus	−33, 24, −8
		2	Anterior insular cortex, adjacent to the inferior frontal gyrus	−34, 19, −7
		3	Anterior insular cortex, first short gyrus	−34, 14, −6
		4	Anterior insular cortex, second short gyrus	−34, 9, −3
		5	Anterior insular cortex, transition between the second and third short gyri	−35, 3, −2
		6	Anterior insular cortex, third short gyrus	−35, 0, 0
		7	Anterior insular cortex, transition between the third short gyrus and the first long gyrus	−35, −6, 1
6	Left	1	Anterior insular cortex, first short gyrus	−35, 14, −1
		2	Anterior insular cortex, adjacent to the opercular portion of the inferior frontal gyrus	−39, 14, −3
		3	Anterior insular cortex, adjacent to the opercular portion of the inferior frontal gyrus	−44, 13, −6
2 (second surgery)	Left	1	Anterior insular cortex, adjacent to the opercular portion of the inferior frontal gyrus	−42, 22, −1
		2	Anterior insular cortex, adjacent to the opercular portion of the inferior frontal gyrus	−42, 16, −1
		3	Anterior insular cortex, second short gyrus	−42, 11, −1
		4	Anterior insular cortex, transition between the second and third short gyri	−42, 4, −1
		5	Anterior insular cortex, third short gyrus	−42, −1, 0
		6	Posterior insular cortex, first long gyrus	−42, −8, 0
		7	Posterior insular cortex, transition between the first and the second long gyri	−42, −15, 0
		8	Posterior insular cortex, second long gyrus	−42, −19, 0

Based on previous studies using scalp EEG and MEG^5,9,11–13,15–18^, we hypothesized that (i) the intensity of stimulation would affect intensity ratings, as well as the magnitudes of both low-frequency phase-locked LFPs and GBOs elicited in the insula; (ii) that stimulus repetition at a constant and predictable ISI would decrease the magnitudes of low-frequency phase-locked LFPs recorded from the human insula, but would not affect intensity ratings nor the magnitudes of GBOs recorded at the same insular locations.

## Results

### Quality and intensity of perception

**Figure 3:**
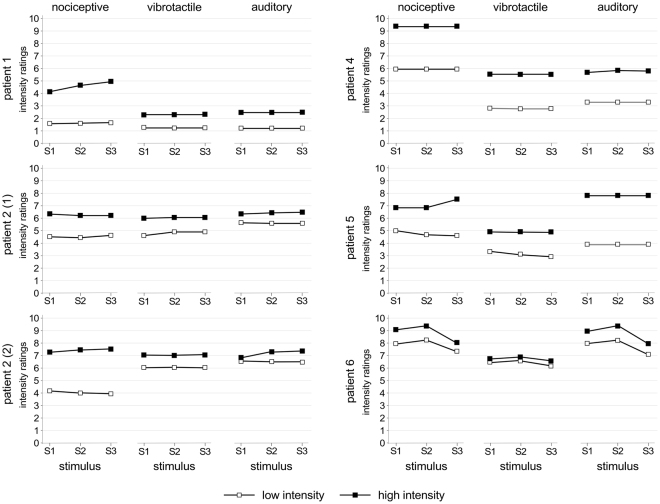
Average ratings of intensity of perception for each participant and modality of stimulation. The ratings of intensity were affected by stimulation intensity: for all three modalities (nociceptive, vibrotactile, and auditory), each participant rated high-intensity stimuli (in black) as more intense than low-intensity stimuli (in white). In contrast, intensity of perception was not affected by stimulus repetition, as participants provided similar ratings for each of the three stimuli belonging to the triplets (note that ratings from one participant were lost due to a hardware failure).

All participants described laser nociceptive stimuli as tolerable, but nevertheless painful and pricking. Non-nociceptive vibrotactile and auditory stimuli were described as clearly perceivable and intense, but never painful. The ratings of intensity (“high” nociceptive: 7.2 ± 1.7; “low” nociceptive: 4.8 ± 2.0; “high” vibrotactile: 5.1 ± 1.6; “low” vibrotactile: 3.7 ± 1.8; “high” auditory: 6.3 ± 2.1; “low” auditory: 4.4 ± 2.2; values expressed as mean ± standard deviation) provided by each participant are shown in Fig. 3. In all three modalities, intensity ratings differed significantly (nociceptive: χ^2^ = 21.2, p = 0.001; vibrotactile: χ^2^ = 20.1, p = 0.001; auditory: χ^2^ = 23.4, p < 0.001). Pairwise comparisons showed that this difference could be explained by an effect of the intensity of stimulation, as participants rated high-intensity stimuli as more intense than low-intensity stimuli (p < 0.001 for all three modalities). However, for all three modalities, the ratings for the first (S1), second (S2), and third (S3) stimuli did not differ significantly (p = 1.000 for all comparisons).

### Low-frequency phase-locked LFPs recorded from the human insula

All types of stimuli elicited low-frequency phase-locked LFPs in the insula, appearing as large biphasic waves (Supplemental Material 1 and 2). A linear mixed model (LMM) analysis performed on the average peak-to-peak amplitude of the low frequency phase-locked LFPs elicited by the triplets, using “modality” (nociceptive, vibrotactile, auditory), “intensity” (high, low), and “stimulus repetition” (S1, S2, S3) as fixed factors, and “subject” as a contextual variable, showed a main effect of “modality” (F = 55.4, p < 0.001), a main effect of “intensity” (F = 23.6, p < 0.001), and a main effect of “stimulus repetition” (F = 77.9, p < 0.001). Post-hoc comparisons showed that both nociceptive and auditory low-frequency phase-locked LFPs were significantly greater in amplitude than vibrotactile low-frequency phase-locked LFPs (both p < 0.001); that low-frequency phase-locked LFPs elicited by high-intensity stimuli were significantly greater in amplitude than low-frequency phase-locked LFPs elicited by low-intensity stimuli; and that low-frequency phase-locked LFPs elicited by the first stimuli of the triplets (S1) were significantly greater in amplitude than low-frequency phase-locked LFPs elicited by the second (S2) and third (S3) stimuli of the triplets.

**Figure 4:**
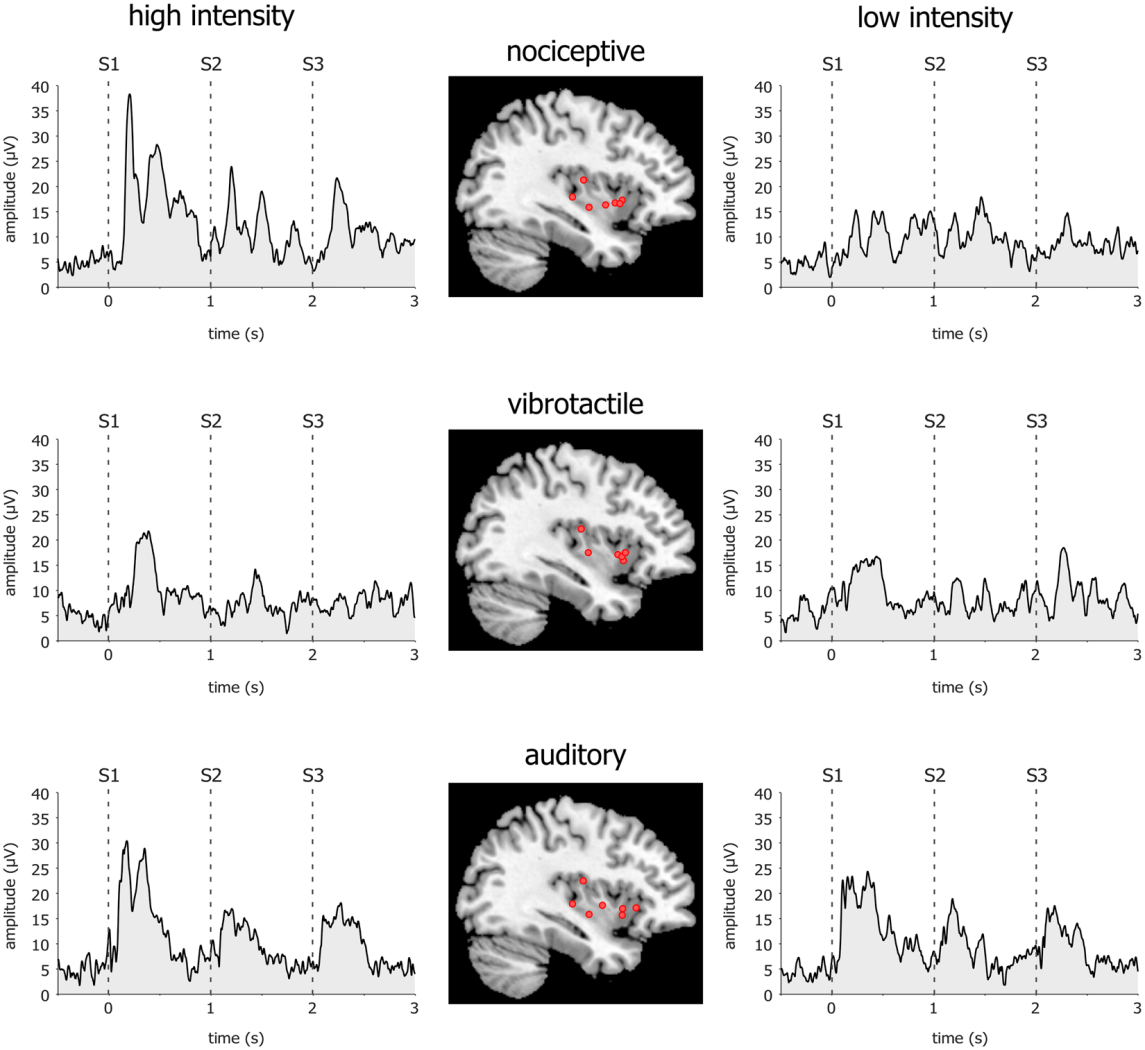
Low-frequency phase-locked local field potentials (LFPs) elicited in the human insula. The waveforms represent rectified low-frequency phase-locked LFPs elicited by high-intensity and low-intensity nociceptive, vibrotactile, and auditory “triplets” recorded at the insular contacts in which, for each explored insula, the amplitude of the response was greatest (group-level average). For each modality, the locations of the selected insular contacts are displayed in the middle panel (red circles). For all three modalities (nociceptive, vibrotactile, auditory), low-frequency phase-locked LFPs elicited by high-intensity stimuli were significantly greater in amplitude than low-frequency phase-locked LFPs elicited by low-intensity stimuli. When stimuli were delivered at high intensity, stimulus repetition led to a significant decrease of the amplitude of low-frequency phase-locked LFPs, for all three modalities. When stimuli were delivered at low intensity, this decrease was only significant for the auditory modality, given the very small amplitude of low-frequency phase-locked LFPs elicited by low-intensity nociceptive and vibrotactile stimuli. Individual low-frequency phase-locked LFPs recorded from all insular contacts in each participant are shown in Supplemental Material 1 and 2.

The analysis also yielded a significant three-way interaction between “modality”, “intensity”, and “stimulus repetition” (Table 2). Post-hoc comparisons showed that, when stimuli were delivered at high intensity, stimulus repetition led to a significant decrease of the amplitude of low-frequency phase-locked LFPs in all three modalities. In contrast, when stimuli were delivered at low intensity, such a decrease was only significant for the auditory modality. Figure 4 shows the rectified low-frequency phase-locked LFPs recorded from the insular contact at which the response amplitude was greatest, averaged across participants.

**Table 2:** Pairwise comparisons of the amplitudes of low-frequency phase-locked local field potentials elicited by the first stimuli of the triplets (S1) and the following stimuli (S2, S3) in the nociceptive, vibrotactile, and auditory modalities, at high and low intensities.

		S1 vs S2		S1 vs S3	
		mean difference (μV)	p-value	mean difference (μV)	p-value
nociceptive	high	18	**p < 0.001** ^*^	29	**p < 0.001** ^*^
	low	3	p = 0.933	1	p = 1
vibrotactile	high	7	p = 0.098	14	**p < 0.001** ^*^
	low	5	p = 0.353	2	p = 1
auditory	high	**25**	**p < 0.001** ^*^	25	**p < 0.001** ^*^
	low	**21**	**p < 0.001** ^*^	24	**p < 0.001** ^*^

### GBOs recorded from the human insula

**Figure 5:**
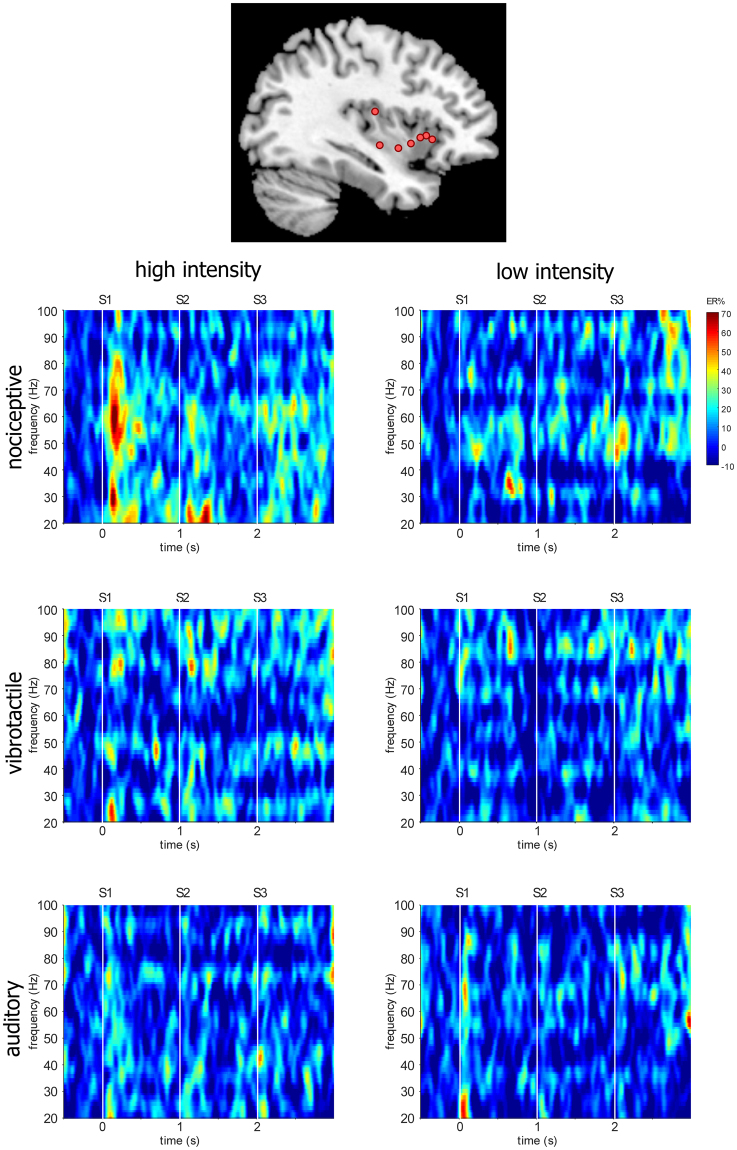
Nociceptive gamma-band oscillations (GBOs) elicited in the human insula. Time-frequency representation of the changes in oscillatory power elicited by nociceptive, vibrotactile, and auditory “triplets”, at the insular contacts in which, for each explored insula, GBOs elicited by nociceptive stimuli were more pronounced (group-level average percentage change in magnitude: ER%). The locations of the selected insular contacts are displayed on the upper panel (red circles). As compared to non-nociceptive stimuli, nociceptive stimuli elicited a greater post-stimulus increase in GBO power, particularly in the 40–90 Hz frequency range. The enhancement of nociceptive GBOs following the first stimuli of the triplets (S1) was significantly greater in magnitude than the enhancement of nociceptive GBOs following the second (S2) and third (S3) stimuli of the triplets. This enhancement was also significantly greater following high-intensity nociceptive stimuli compared to low-intensity nociceptive stimuli. Individual time-frequency maps from each participant are available at the OSF online repository at the address www.osf.io/yw4nf.

Nociceptive stimuli elicited an enhancement of GBOs at several insular contacts, which was not observed in response to non-nociceptive vibrotactile, auditory, and visual stimuli (Fig. 5). A LMM analysis performed on the average event-related percentage of change in signal amplitude (ER%) between 150 and 300 ms after each stimulus^8^, using factors “modality” (nociceptive, vibrotactile, auditory), “intensity” (high, low), “stimulus repetition” (S1, S2, S3), and “frequency range” (40–90 Hz, 90–140 Hz) as fixed factors, and “subject” as a contextual variable, showed a significant effect of “modality” (F = 21.32, p < 0.001). On average, the magnitudes of nociceptive GBOs were significantly greater than the magnitudes of vibrotactile and auditory GBOs (all p < 0.001). The analysis also showed a significant interaction between “intensity” and “frequency range” (F = 6.81, p = 0.009). Post-hoc comparisons showed that in the 40–90 Hz frequency range, GBOs elicited by high-intensity stimuli were significantly greater in magnitude than GBOs elicited by low-intensity stimuli. This was not the case for higher-frequency activities elicited in the 90–140 Hz frequency range. Moreover, the analysis showed a significant three-way interaction between “modality”, “stimulus repetition”, and “intensity” (F = 2.90, p = 0.021). Post-hoc comparisons showed that nociceptive GBOs elicited by high-intensity stimuli were significantly greater in magnitude than GBOs elicited by low-intensity stimuli following S1 (p < 0.001) and S2 (p = 0.002), but not following S3 (p = 0.083). Finally, the analysis showed a significant three-way interaction between “modality”, “stimulus repetition”, and “frequency range” (F = 2.82, p = 0.024). In the 40–90 Hz frequency range, but not in the 90–140 frequency range, nociceptive GBOs were affected by stimulus repetition, as GBOs elicited by S1 were significantly greater in magnitude than GBOs elicited by S2 (p = 0.001) and S3 (p = 0.016).

## Discussion

Consistently with previous studies^1,8^, all three types of stimuli (nociceptive, vibrotactile, auditory) elicited robust low-frequency phase-locked LFPs in the insula. In addition to these low-frequency responses, nociceptive stimuli, but not non-nociceptive vibrotactile and auditory stimuli, elicited a marked enhancement of GBOs at the same insular locations. This result is consistent with our recent finding that GBOs preferential for nociception can be recorded from the human insula^8^, and that low-frequency phase-locked LFPs are dissociated from GBOs recorded at the same insular locations.

Stimulation intensity modulated the intensity of perception, as well as the magnitude of both low-frequency phase-locked LFPs and nociceptive GBOs recorded from the human insula. In contrast, stimulus repetition at a constant and predictable 1-s ISI had no effect on the intensity of perception, while leading to a decrease in magnitude of both low-frequency phase-locked LFPs and nociceptive GBOs (40–90 Hz). These results show that the magnitudes of insular low-frequency phase-locked LFPs and nociceptive GBOs can be dissociated from the intensity of perception – a dissociation that is consistent with several findings in the field of perceptual neuroscience, which suggest that a significant portion of the brain activity evoked by a brief sensory stimulus is not directly related to the quality and the intensity of the corresponding sensation, but could be related to bottom-up attentional processes^19–22^.

In our study, high-intensity triplets led to a habituation of low-frequency phase-locked LFPs in all three modalities. In contrast, low-intensity triplets led to a habituation of low-frequency phase-locked LFPs in the auditory modality, but not in the nociceptive and vibrotactile modalities. This difference could be explained by a floor effect due to the much smaller responses elicited by low-intensity nociceptive and vibrotactile stimuli. In agreement with our findings, several scalp EEG and MEG studies have shown that the repeated presentation of a stimulus at a constant ISI leads to a habituation of the stimulus-evoked brain responses^5,9–17,23^. These studies indicate that habituation takes place regardless of the modality of stimulation, as it was observed for thermonociceptive stimuli^5,9,11–13,15–17^, as well as for innocuous somatosensory^11,14^ and auditory^10,14,16,23^ stimuli.

Some researchers initially interpreted the response decrement induced by stimulus repetition as a consequence of neuronal or psychological *refractoriness*^11,24,25^, but these interpretations have been ruled out by a number of studies showing that (i) when stimuli are repeated at an *unpredictable* ISI, stimulus repetition does not lead to a response decrement^10,12,14,15,23^; (ii) the response decrement is not reflected in the perceived intensity of the stimulus^13,18^; and (iii) the duration of neuronal refractoriness has been estimated to last only a few milliseconds, whereas the response decrement is observed at longer ISIs^13,26^.

Because a stimulus that is repeated in time stands out less in the sensory environment, and because a short and constant ISI makes the stimulus more predictable, stimulus *saliency*, rather than neuronal or psychological refractoriness, may better explain the reduction in the amplitude of brain responses when a stimulus is repeated at a short and constant ISI^5,13,15,16^. In the present experiment, the first stimulus of each triplet (S1) was preceded by the last stimulus of the previous triplet (S3) by a long, variable, and unpredictable interval (10–15 s). In contrast, a constant interval of 1 s separated the onset of the second (S2) and third (S3) stimuli from the onset of the preceding stimulus, making S2 and S3 more predictable than S1. In addition, whereas the intensity of stimulation (“high” or “low”) randomly varied from triplet to triplet, it was constant *within* each triplet. Hence, the intensity of S1 predicted the intensity of S2 and S3, but the intensity of S3 did not predict the intensity of the first stimulus (S1) of the following triplet. Because both the time of occurrence and the intensity of stimulation of S1 were more unexpected than the time of occurrence and the intensity of stimulation of S2 and S3, it is reasonable to assume that S1 was more salient than S2 and S3 – a difference that could explain the observed decrease in the magnitude of the elicited low-frequency phase-locked LFPs and GBOs with stimulus repetition.

A stimulus perceived as intense is likely more salient than a stimulus perceived as weak^27^. Hence, the relationship between the magnitude of the stimulus-evoked insular responses and the intensity of stimulation could also be explained by an effect of stimulus saliency on response magnitude. The saliency hypothesis is also in line with the proposal that the insula belongs to a cortical network involved in the detection of significant events that are potentially threatening for the body, acting as a defensive system, regardless of the sensory channel through which these events are conveyed^7^. Notably, the insula was suggested to constitute a hub connecting sensory areas to other networks involved in the processing and integration of external and internal information, allowing to gain a coherent representation of different salient conditions, including, but not limited to, pain-related experiences^6,28,29^.

A slightly different explanation for the habituation of the stimulus-evoked responses could be the lack of novelty of the second (S2) and third (S3) stimuli of the triplet compared to the first stimulus (S1), rather than reduced saliency. Using scalp EEG, Ronga and collaborators^5^ showed that varying the intensity of the third stimulus of a triplet led to a dishabituation of the nociceptive-evoked response when the intensity was increased, but not when it was decreased. This study indicates that the magnitude of nociceptive ERPs is mainly determined by stimulus saliency, rather than stimulus novelty. Further investigations with a similar paradigm using intracerebral EEG could test whether this is also the case for low-frequency phase-locked LFPs and nociceptive GBOs recorded from the human insula.

Whereas thermal nociceptive stimuli selectively activate the nociceptive system^30^, low-frequency phase-locked LFPs recorded from the insula mostly reflect neural processes that are not selective or specific for the nociceptive system, but are possibly triggered by any salient stimulus occurring in the environment, regardless of its sensory modality. Compared to low-frequency phase-locked LFPs, GBOs (40–90 Hz) elicited in the human insula by nociceptive stimuli appear to be more selective for nociception and/or activation of the spinothalamic system, because they are not elicited by non-nociceptive vibrotactile and auditory stimuli.

The most remarkable and unexpected finding of our study is that nociceptive GBOs recorded from the human insula are strongly affected by stimulus repetition. This result suggests that these responses could at least partly reflect neural processes related to the processing of salient events. The main implication of this finding is that nociceptive GBOs recorded from the human insula cannot be considered as a direct correlate of pain perception.

The observation that stimulus repetition induces a marked habituation of nociceptive GBOs recorded from the human insula appears in contradiction with the results of Zhang and collaborators^18^, who reported that the magnitudes of GBOs recorded using scalp EEG and hypothesized to originate from the contralateral SI correlate with the intensity of pain perception independently of stimulus repetition. The authors concluded that these GBOs constitute a “direct obligatory correlate of subjective pain intensity”. A possible explanation for the divergence between our results and the results of Zhang *et al*.^18^ is that GBOs recorded over SI using scalp EEG and GBOs recorded from the insula using intracerebral EEG reflect functionally distinct cortical processes. Whereas GBOs recorded over SI might more closely relate to subjective pain intensity and/or the strength of the eliciting stimulus, the latter appear to be strongly related the saliency of the eliciting stimuli, while still being preferential for nociceptive and/or spinothalamic input.

The fact that nociceptive GBOs recorded over different brain regions appear to reflect functionally distinct processes indicates that the relationship between these activities and pain perception should always be interpreted with caution. Crucially, because pain and nociception are often related, but can occur independently of each other, nociceptive brain activity can only be considered as a proxy measure of pain^7,31^. For instance, sleep deprivation was shown to decrease the amplitude of brain responses elicited by nociceptive stimuli, while causing hyperalgesia^32–34^. Nociceptive brain responses can be detected during sleep^35^, in anaesthesized patients^36^, and in patients with disorders of consciousness^37,38^, yet in the absence of behavioral evidence suggesting a conscious experience of pain. In a number of other pathological conditions, nociceptive brain responses can be absent or reduced, despite preserved or enhanced pain perception^39–44^. In other conditions, normal nociceptive brain responses can be associated to reduced pain perception^45^. Finally, numerous studies have shown that the physical intensity of the nociceptive stimuli, the magnitude of the nociceptive brain responses, and the subjective intensity of pain, can be modulated independently^46–50^, particularly when attentional or emotional processes are at play^21,48^.

Similarly to brain responses elicited by nociceptive stimuli, several other nociceptive physiological responses, such as the skin conductance response (SCR)^51–55^, pupil dilation^56–59^, heart rate variability^60–68^, and the nociceptive flexion reflex (NFR)^69–72^, are often used to objectify the experience of pain in both animals and humans. In humans, the magnitude of these physiological responses can, in some circumstances, correlate reliably with self-reported pain – the current “gold standard” in pain assessment^73,74^. Notwithstanding, just as for nociceptive brain responses, these other physiological responses may also be dissociated from perceived pain intensity^73–79^. For instance, Bromm and Scharein^73^ demonstrated that when an electrical nociceptive stimulus is repeated at a variable ISI (20–40 s), neither pain perception nor event-related potentials are affected by stimulus repetition, whereas the SCR, the electrooculogram (EOG), and the NFR all show a marked decrease in amplitude. Rhudy and collaborators^75^ reported that the repetition of an electrical nociceptive stimulus leads to a habituation of the SCR and NFR, whereas pain ratings increase. These findings suggest that different spinal and supraspinal responses can be differently affected by stimulus repetition, leading to dissociated patterns of response decrement or increment to the same stimulus.

Animal studies have shown that the repetition of a nociceptive stimulus can induce either habituation or sensitization of the response of spinal dorsal horn neurons, depending on the intensity and frequency of stimulation^80–82^. Most interestingly, different effects of stimulus repetition have been observed in different types of spinal dorsal horn neurons responding to nociceptive input. Interneurons displaying only habituation patterns, found in laminae I-V, were found to be anatomically distinct from interneurons displaying a sensitization-habituation pattern, found in laminae V-VII^80^. Hence, habituation and sensitization appear to be independently mediated by different neuronal circuits^81^. Moreover, Ikeda and collaborators^83^ showed that electrical low-frequency stimulation (LFS, 2 Hz) and high-frequency stimulation (HFS, 100 Hz) of C-fiber primary afferents exert different effects on synaptic transmission in spinal neurons projecting to the parabrachial (PB) area compared to spinal neurons projecting to the periaqueductal gray (PAG). HFS, but not LFS, induced long-term potentiation (LTP) at synapses between C fibers and neurons projecting to the PB area. In contrast, LFS, but not HFS, induced LTP at synapses between C fibers and neurons projecting to the PAG. In other words, synaptic plasticity appears to be differentially induced in distinct ascending nociceptive tracts. These observations raise the possibility that differential effects of stimulus repetition on pain perception and nociceptive brain responses could be due, at least in part, to a differential effect on different populations of dorsal horn neurons conveying nociceptive input to the brain.

In conclusion, the fact that stimulus repetition leads to a dissociation between the intensity of pain perception and the magnitude of both nociceptive low-frequency phase-locked LFPs and high-frequency GBOs recorded from the human insula indicates that these responses are not directly related to the “encoding” of pain intensity. Therefore, and despite the fact that they appear to reflect cortical activity selective for thermonociception, insular nociceptive GBOs cannot be considered as a correlate of perceived pain.

## Materials and Methods

### Participants

Six patients (3 females, mean age: 32, range: 23–51) suffering from intractable focal epilepsy were recruited at the Department of Neurology of the Saint Luc University Hospital (Brussels, Belgium). All patients were investigated using depth electrodes implanted in various brain regions suspected to be the origins of seizures, including wide portions of the anterior and posterior insula (Fig. 1; see Table 1 for the MNI coordinates of each electrode contact). The intracerebral EEG was recorded from 8 electrodes located in the insula (Patient 2 underwent a second electrode implantation after 18 months, encompassing additional insular locations; Patient 4 had two distinct insular electrodes implanted concomitantly, one in the dorsal posterior insula/operculum and one in the ventral posterior insula), for a total of 46 insular sites. Because 5 contacts were excluded from the analysis due to strong electrical artifacts of unknown origin, electrophysiological responses from 41 insular contacts were investigated. The MNI coordinates and the description of the location of each contact are presented in Table 1. In addition, the anonymized DICOM images of each patient are available at the OSF online repository at the address www.osf.io/yw4nf. These images do not include facial features that could lead to the identification of the participants.

None of the participants had psychiatric issues, cognitive impairment, or sensory abnormalities. None of the participants presented ictal discharge onset in the insula during the recordings, and low-voltage fast activity was never present in this area during spontaneous seizures. Four of the patients (Patients 1–4) also participated in two previous studies conducted by our team^1,8^. All participants gave written informed consent. All experimental procedures were approved by the local Research Ethics Committee (Commission Ethique de l’Université catholique de Louvain, B403201316436) and carried out in accordance with the relevant guidelines and regulations. The manuscript does not contain information or images that could lead to the identification of the participants.

### Procedure

The study was conducted at the patient bedside. Before the beginning of the experiment, the procedure was explained to the patient, who was exposed to a small number of test stimuli for familiarization. The experiment consisted of three blocks (Fig. 2). In each block, participants received stimuli from one of three sensory modalities: nociceptive, vibrotactile, and auditory. The stimuli were delivered in trains of three stimuli (S1-S2-S3, a “triplet”). Within each triplet, stimuli had identical intensity, and were delivered at a constant ISI of 1 s. The time interval between triplets was variable and self-paced by the experimenter (10–15 s) to avoid expectation. Two different intensities, “high” and “low”, were used for each modality. “High” and “low” triplets were delivered in a randomized order within the blocks. Participants were instructed to fixate a black cross (3 × 3 cm) placed in front of them, at a distance of ~2 m, 30° below eye level, for the whole duration of each block. Because Iannetti and collaborators^13^ have shown that subjects are able to independently and reliably rate the intensity of three consecutive stimuli presented at a 1-s ISI, we asked the participants to verbally rate, *at the end of each triplet*, the intensity of each stimulus of the triplet on a numerical scale ranging from 0 to 10, where “0” was defined as “no sensation at all”, and “10 was defined as “highest intensity imaginable” (ratings from one participant were lost due to a computer failure). As the time interval between triplets was self-paced by the experimenter, participants had enough time to provide the ratings without excessive time pressure. Participants were asked for ratings of *intensity* rather than ratings of a modality-specific perceptual feature such “painfulness” or “loudness” to keep the task consistent across the different blocks. Finally, at the end of each block, participants were asked to report whether they had perceived the stimuli as painful. The order of the blocks was randomized across participants. Due to health issues, Patient 3 only participated to the nociceptive and auditory blocks, but interrupted the experiment before undergoing the vibrotactile block.

### Sensory stimuli

Nociceptive stimuli consisted of 4-ms pulses of radiant heat generated by an infrared neodymium yttrium aluminum perovskite laser with a wave-length of 1.34 μm (Nd:YAP, Electronical Engineering, Florence, Italy), applied on the hand dorsum contralateral to the implanted insular electrode. The laser beam diameter was set at 5 mm by focusing lenses. Stimulation intensity was 1.25 J for “low” nociceptive stimuli and 1.50 J for “high” nociceptive stimuli. These intensities, fixed ahead of time, were chosen following pilot tests performed on healthy subjects, to ensure that the stimuli would always be perceived as clearly painful and pricking – a sensation shown to be related to the activation of Aδ fiber skin nociceptors^30^ – while still being easily discriminated in terms of intensity. To prevent nociceptor fatigue or sensitization, the laser beam was manually displaced by about 2 cm after each stimulus^84^.

Vibrotactile stimuli consisted in 50-ms vibrations at 245 Hz, delivered via a recoil-type vibrotactile transducer driven by a standard audio amplifier (Haptuator, Tactile Labs Inc., Canada) and positioned on the palmar side of the index fingertip contralateral to the implanted insular electrode. Auditory stimuli were brief 800-Hz lateralized tones (50 ms duration; 0.5 left/right amplitude ratio), delivered through an earphone contralateral to the implanted insular electrode. Stimulation volume was 92 dB SPL for “low” auditory stimuli and 99 dB SPL for “high” auditory stimuli. The intensities of non-nociceptive vibrotactile and auditory stimuli were chosen after pilot tests performed on healthy subjects, to ensure that all stimuli would be clearly perceived and discriminated in terms of intensity, without eliciting a painful sensation.

### Analysis of the intensity of perception

To investigate whether stimulation intensity and stimulus repetition had an effect on the intensity of perception, we performed, for each modality of stimulation, a Friedman test. Main effects were compared using the Bonferroni confidence interval adjustment.

### Intracerebral recordings and anatomical electrode contact localization

For each patient, a tailored implantation strategy was planned according to the regions considered most likely to be ictal onset sites or propagation sites, as described in previous works from our group^1,8^. Target areas, including the insular cortex, were reached using commercially available depth electrodes (AdTech, Racine, WI, U.S.A.; contact length: 2.4 mm; contact spacing: 5 mm) implanted using a frameless stereotactic technique through burr holes. The placement was guided by a neuronavigation system based on 3D-T1W magnetic resonance imaging (MRI) sequence performed in a 1.5 T scanner (Gradient Echo; flip angle: 15°; TR: 7.5 s; TE minimum full; 3.1–13 ms; slice thickness: 1 mm; FOV: 24 cm; matrix: 224 × 224; number of slices: 162). A post-implantation 3D-T1 MRI sequence, performed either right after the surgery or on the following day, was used to accurately identify the locations of each electrode contact. This acquisition mode is safe and compatible with implanted electrodes, which can be clearly identified when analyzed with a 3D-T1W sequence after gadolinium injection. To obtain the MNI coordinates of each insular electrode contact, individual MRI scans were normalized to a standard T1 template in MNI space using BrainVoyager 20.2 (Brain Innovation, Maastricht, The Netherlands).

The intracerebral EEG recordings were performed using a DeltaMed (Paris, France) acquisition system. Additional bipolar channels were used to record electromyographic activity (EMG: two electrodes measuring respectively bicipital and tricipital contraction of the patient’s non-dominant arm) and electrocardiographic activity (EKG: two channels, utilizing two electrodes respectively located on the right and left side of the sternum, one electrode located centrally under the sternum, and one electrode on the right lateral side of the chest). All signals were acquired at a 512 Hz sampling rate using a reference electrode located between Cz and Pz, re-referenced to the average activity recorded from all intracerebral contacts, and analyzed offline using Letswave 6 (http://nocions.org/letswave)^85^. Additional statistical analyses were carried out using IBM SPSS Statistics 24 (Armonk, NY).

### Analysis in the time domain

For the analysis in the time domain, the continuous recordings were filtered using a Butterworth band-pass filter (0.3–40 Hz). Epochs were obtained by segmenting the recordings from −0.5 to 3 s relative to the onset of each “triplet”. Trials contaminated by artefacts were corrected using an independent component analysis (ICA) algorithm^86^. Separate average waveforms were computed for each subject, stimulus type (nociceptive, vibrotactile, auditory), and stimulus intensity (high, low). Baseline subtraction was performed using the reference interval between −0.5 and 0 s relative to the first stimulus of each triplet. Within the averaged waveforms, the peak-to-peak amplitudes of the large biphasic waves elicited by each stimulus were used as a measure of the amplitude of the stimulus-evoked LFPs.

A linear mixed model (LMM) analysis was performed on the averaged amplitudes of the phase-locked LFPs using the factors “modality” (3 levels: nociceptive, vibrotactile, auditory), “stimulus intensity” (2 levels: low, high), and “stimulus repetition” (3 levels: S1, S2, S3). The contextual variable “subject” was added to the model, to account for the variation of the regression model intercept across participants. Parameters were estimated using restricted maximum likelihood (REML)^87^. Main effects were compared using the Bonferroni confidence interval adjustment.

### Analysis in the time-frequency domain

For the analysis in the time-frequency domain, the continuous recordings were filtered using a high-pass Butterworth filter (>20 Hz). Epochs were obtained by segmenting the recordings from −0.5 to 3 s relative to the onset of each triplet. Trials contaminated by artefacts were corrected using ICA. A time-frequency representation of each high-pass filtered epoch was obtained using a short-term Fourier transform (STFT) with a fixed 200-ms width Hanning window, chosen to achieve a good tradeoff between time resolution and frequency resolution in the range of gamma-band frequencies^18,88,89^. The STFT yielded, for each trial, a complex time-frequency spectral estimate F(t, f) at each point (t, f) of the time-frequency domain plane extending from −0.5 to 1 s in the time domain, and from 20 to 150 Hz (in steps of 1 Hz) in the frequency domain. After averaging the single-trial time-frequency maps for each subject, stimulus type (nociceptive, vibrotactile, auditory), and stimulus intensity (high, low), the average magnitude of the stimulus-induced changes in oscillation amplitude was estimated as follows^18,90,91^: *ER*%(*t*, *f*) = [*P*(*t*, *f*) − *R*(*f*)]/*R*(*F*) × 100; where P(t, f) = |F(t, f)|^2^ is an estimate of signal amplitude at each time-frequency point (t, f) and R(f) is the average amplitude of the signal enclosed within the prestimulus reference interval (−0.4 to −0.1 s before the onset of the stimulus), for each estimated frequency, f. This yielded, for each insular electrode contact, modality of stimulation, and intensity of stimulation, a time–frequency representation of the average stimulus-induced changes of intracerebral EEG signal (event-related percentage of change in signal amplitude, ER%)^90^

A LMM analysis was performed on the average event-related percentage of change in signal power within the 150–300 ms post-stimulus interval^8^, using the factors “modality” (3 levels: nociceptive, vibrotactile, auditory), “stimulus intensity” (2 levels: low, high), “stimulus repetition” (3 levels: S1, S2, S3), and “frequency range” (2 levels: 40–90 Hz, 90–140 Hz). This last factor was included given our recent observation that, although thermal nociceptive stimuli elicit insular GBOs predominantly in the 40–90 Hz frequency range, an enhancement of gamma-band activity can be found also at higher frequencies^8^. The contextual variable “subject” was added to the model, to account for the variation of the regression model intercept across participants. Parameters were estimated using REML^87^. Main effects were compared using the Bonferroni confidence interval adjustment.

### Data availability

All supporting data is available at the OSF online repository at the address www.osf.io/yw4nf.

## Electronic supplementary material

Supplemental material

## References

[1] Liberati G (2016). Nociceptive Local Field Potentials Recorded from the Human Insula Are Not Specific for Nociception. PLoS Biol..

[2] Frot M, Magnin M, Mauguière F, Garcia-Larrea L (2007). Human SII and posterior insula differently encode thermal laser stimuli. Cereb. Cortex.

[3] Frot M, Mauguière F (2003). Dual representation of pain in the operculo-insular cortex in humans. Brain.

[4] Bastuji H, Frot M, Perchet C, Magnin M, Garcia-Larrea L (2016). Pain networks from the inside: Spatiotemporal analysis of brain responses leading from nociception to conscious perception. Hum. Brain Mapp..

[5] Ronga I, Valentini E, Mouraux A, Iannetti GD (2013). Novelty is not enough: laser-evoked potentials are determined by stimulus saliency, not absolute novelty. J. Neurophysiol..

[6] Yantis S (2008). The Neural Basis of Selective Attention: Cortical Sources and Targets of Attentional Modulation. Curr Dir Psychol Sci.

[7] Legrain V, Iannetti GD, Plaghki L, Mouraux A (2011). The pain matrix reloaded: a salience detection system for the body. Prog. Neurobiol..

[8] Liberati, G. *et al*. Gamma-Band Oscillations Preferential for Nociception can be Recorded in the Human Insula. *Cereb*. *Cortex* 1–15 10.1093/cercor/bhx237 (2017). 10.1093/cercor/bhx237 PMC6366557 29028955

[9] Bromm B, Treede RD (1987). Human cerebral potentials evoked by CO2 laser stimuli causing pain. Exp. Brain Res..

[10] Loveless N, Hari R (1989). Ha¨ma¨la¨inen, M. & Tiihonen, J. Evoked responses of human auditory cortex may be enhanced by preceding stimuli. Electroencephalography and Clinical Neurophysiology/Evoked Potentials Section.

[11] Truini A (2004). Excitability of the Adelta nociceptive pathways as assessed by the recovery cycle of laser evoked potentials in humans. Exp. Brain Res..

[12] Mouraux A, Guérit JM, Plaghki L (2004). Refractoriness cannot explain why C-fiber laser-evoked brain potentials are recorded only if concomitant Adelta-fiber activation is avoided. Pain.

[13] Iannetti GD, Hughes NP, Lee MC, Mouraux A (2008). Determinants of laser-evoked EEG responses: pain perception or stimulus saliency?. J. Neurophysiol..

[14] Wang AL, Mouraux A, Liang M, Iannetti GD (2008). The enhancement of the N1 wave elicited by sensory stimuli presented at very short inter-stimulus intervals is a general feature across sensory systems. PLoS One.

[15] Wang AL, Mouraux A, Liang M, Iannetti GD (2010). Stimulus novelty, and not neural refractoriness, explains the repetition suppression of laser-evoked potentials. J. Neurophysiol..

[16] Valentini E, Torta DME, Mouraux A, Iannetti GD (2011). Dishabituation of laser-evoked EEG responses: dissecting the effect of certain and uncertain changes in stimulus modality. J. Cogn. Neurosci..

[17] Torta DM, Liang M, Valentini E, Mouraux A, Iannetti GD (2012). Dishabituation of laser-evoked EEG responses: dissecting the effect of certain and uncertain changes in stimulus spatial location. Exp. Brain Res..

[18] Zhang ZG, Hu L, Hung YS, Mouraux A, Iannetti GD (2012). Gamma-band oscillations in the primary somatosensory cortex–a direct and obligatory correlate of subjective pain intensity. J. Neurosci..

[19] Chapman CR, Colpitts YH, Mayeno JK, Gagliardi GJ (1981). Rate of stimulus repetition changes evoked potential amplitude: dental and auditory modalities compared. Exp. Brain Res..

[20] Dowman R (1996). Effects of interstimulus interval on scalp topographies evoked by noxious sural nerve stimulation. Psychophysiology.

[21] Kulkarni B (2005). Attention to pain localization and unpleasantness discriminates the functions of the medial and lateral pain systems. Eur. J. Neurosci..

[22] Seminowicz DA, Davis KD (2007). Interactions of pain intensity and cognitive load: the brain stays on task. Cereb. Cortex.

[23] Budd TW, Michie PT (1994). Facilitation of the N1 peak of the auditory ERP at short stimulus intervals. Neuroreport.

[24] Truini A, Galeotti F, Cruccu G, Garcia-Larrea L (2007). Inhibition of cortical responses to Adelta inputs by a preceding C-related response: testing the “first come, first served” hypothesis of cortical laser evoked potentials. Pain.

[25] Pashler H (1984). Processing stages in overlapping tasks: Evidence for a central bottleneck. Journal of Experimental Psychology: Human Perception and Performance.

[26] Hodgkin AL, Huxley AF (1952). A quantitative description of membrane current and its application to conduction and excitation in nerve. J. Physiol. (Lond).

[27] Downar J, Crawley AP, Mikulis DJ, Davis KD (2000). A multimodal cortical network for the detection of changes in the sensory environment. Nat. Neurosci..

[28] Menon V, Uddin LQ (2010). Saliency, switching, attention and control: a network model of insula function. Brain Struct. Funct..

[29] Ranganath C, Rainer G (2003). Neural mechanisms for detecting and remembering novel events. Nat. Rev. Neurosci..

[30] Bromm B, Treede RD (1984). Nerve fibre discharges, cerebral potentials and sensations induced by CO2 laser stimulation. Hum Neurobiol.

[31] Davis KD (2017). Brain imaging tests for chronic pain: medical, legal and ethical issues and recommendations. Nat. Rev. Neurol..

[32] Ødegård SS (2015). The effect of sleep restriction on laser evoked potentials, thermal sensory and pain thresholds and suprathreshold pain in healthy subjects. Clin. Neurophysiol..

[33] Schuh-Hofer S, Baumgärtner U, Treede RD (2015). Effect of sleep deprivation on the electrophysiological signature of habituation to noxious laser stimuli. Eur. J. Pain..

[34] Tiede W (2010). Sleep restriction attenuates amplitudes and attentional modulation of pain-related evoked potentials, but augments pain ratings in healthy volunteers. Pain.

[35] Bastuji H, Perchet C, Legrain V, Montes C, Garcia-Larrea L (2008). Laser evoked responses to painful stimulation persist during sleep and predict subsequent arousals. Pain.

[36] Ní Mhuircheartaigh R, Warnaby C, Rogers R, Jbabdi S, Tracey I (2013). Slow-wave activity saturation and thalamocortical isolation during propofol anesthesia in humans. Sci Transl Med.

[37] Boly M (2008). Perception of pain in the minimally conscious state with PET activation: an observational study. Lancet Neurol..

[38] Kassubek J (2003). Activation of a residual cortical network during painful stimulation in long-term postanoxic vegetative state: a 15O-H2O PET study. J. Neurol. Sci..

[39] Truini A, Cruccu G (2008). Laser evoked potentials in patients with trigeminal disease: the absence of Adelta potentials does not unmask C-fibre potentials. Clin. Neurophysiol..

[40] Ragazzoni A (1993). Electric and CO2 laser SEPs in a patient with asymptomatic syringomyelia. Electroencephalogr. Clin. Neurophysiol..

[41] Simone IL (2010). Laser evoked potentials in amyotrophic lateral sclerosis. J. Neurol. Sci..

[42] De Tommaso M (2011). Nociceptive inputs transmission in Huntington’s disease: a study by laser evoked potentials. Acta Neurol. Belg..

[43] Zambito-Marsala S (2017). Abnormal nociceptive processing occurs centrally and not peripherally in pain-free Parkinson disease patients: A study with laser-evoked potentials. Parkinsonism Relat. Disord..

[44] Wu Q (1999). Hyperalgesia with reduced laser evoked potentials in neuropathic pain. Pain.

[45] Schmahl C (2004). Differential nociceptive deficits in patients with borderline personality disorder and self-injurious behavior: laser-evoked potentials, spatial discrimination of noxious stimuli, and pain ratings. Pain.

[46] van den Broeke EN (2012). The effect of high-frequency conditioning stimulation of human skin on reported pain intensity and event-related potentials. J. Neurophysiol..

[47] Clark JA, Brown CA, Jones AKP, El-Deredy W (2008). Dissociating nociceptive modulation by the duration of pain anticipation from unpredictability in the timing of pain. Clin. Neurophysiol..

[48] Dillmann J, Miltner WH, Weiss T (2000). The influence of semantic priming on event-related potentials to painful laser-heat stimuli in humans. Neurosci. Lett..

[49] Mouraux A, Plaghki L (2007). Cortical interactions and integration of nociceptive and non-nociceptive somatosensory inputs in humans. Neuroscience.

[50] Becerra LR (1999). Human brain activation under controlled thermal stimulation and habituation to noxious heat: an fMRI study. Magn. Reson. Med..

[51] Storm H (2008). Changes in skin conductance as a tool to monitor nociceptive stimulation and pain. Curr. Opin. Anaesthesiol..

[52] Ledowski T (2007). The assessment of postoperative pain by monitoring skin conductance: results of a prospective study. Anaesthesia.

[53] Hullett B (2009). Monitoring electrical skin conductance: a tool for the assessment of postoperative pain in children?. Anesthesiology.

[54] Choo EK (2010). Skin conductance fluctuations correlate poorly with postoperative self-report pain measures in school-aged children. Anesthesiology.

[55] Loggia ML, Juneau M, Bushnell MC (2011). Autonomic responses to heat pain: Heart rate, skin conductance, and their relation to verbal ratings and stimulus intensity. Pain.

[56] Guglielminotti, J. *et al*. Assessment of Pain During Labor with Pupillometry: A Prospective Observational Study. *Anesth*. *Analg*. 10.1213/ANE.0b013e31828a7218 (2013). 10.1213/ANE.0b013e31828a7218 PMC3989209 23477963

[57] Paulus J (2013). Pupillary reflex measurement predict insufficient analgesia before endotracheal suctioning in critically ill patients. Crit. Care.

[58] Aissou M (2012). Objective assessment of the immediate postoperative analgesia using pupillary reflex measurement: a prospective and observational study. Anesthesiology.

[59] Kantor, E., Montravers, P., Longrois, D. & Guglielminotti, J. Pain assessment in the postanaesthesia care unit using pupillometry: A cross-sectional study after standard anaesthetic care. *Eur*. *J*. *Anaesthesiol*. 10.1097/01.EJA.0000434966.96165.c9 (2013). 10.1097/01.EJA.0000434966.96165.c9 24225726

[60] Jeanne M, Logier R, De Jonckheere J, Tavernier B (2009). Heart rate variability during total intravenous anesthesia: effects of nociception and analgesia. Auton Neurosci.

[61] Koenig J, Jarczok MN, Ellis RJ, Hillecke TK, Thayer JF (2014). Heart rate variability and experimentally induced pain in healthy adults: a systematic review. Eur. J. Pain..

[62] Jeanne M, Clément C, De Jonckheere J, Logier R, Tavernier B (2012). Variations of the analgesia nociception index during general anaesthesia for laparoscopic abdominal surgery. J Clin Monit Comput.

[63] Gruenewald M (2013). Influence of nociceptive stimulation on analgesia nociception index (ANI) during propofol-remifentanil anaesthesia. Br. J. Anaesth..

[64] Ledowski T (2013). Analgesia nociception index: evaluation as a new parameter for acute postoperative pain. Br. J. Anaesth..

[65] Ledowski T, Averhoff L, Tiong WS, Lee C (2014). Analgesia Nociception Index (ANI) to predict intraoperative haemodynamic changes: results of a pilot investigation. Acta Anaesthesiol. Scand..

[66] Boselli E (2014). Prediction of immediate postoperative pain using the analgesia/nociception index: a prospective observational study. Br. J. Anaesth..

[67] Boselli, E. *et al*. Prospective observational study of the non-invasive assessment of immediate postoperative pain using the analgesia/nociception index (ANI). *Br*. *J*. *Anaesth*. 10.1093/bja/aet110 (2013). 10.1093/bja/aet110 23592690

[68] Brouse CJ (2013). Monitoring nociception during general anesthesia with cardiorespiratory coherence. J Clin Monit Comput.

[69] Skljarevski V, Ramadan NM (2002). The nociceptive flexion reflex in humans–review article. Pain.

[70] Willer JC (1977). Comparative study of perceived pain and nociceptive flexion reflex in man. Pain.

[71] Arendt-Nielsen L, Brennum J, Sindrup S, Bak P (1994). Electrophysiological and psychophysical quantification of temporal summation in the human nociceptive system. Eur J Appl Physiol Occup Physiol.

[72] García-Larrea L, Sindou M, Mauguière F (1989). Clinical use of nociceptive flexion reflex recording in the evaluation of functional neurosurgical procedures. Acta Neurochir. Suppl. (Wien)..

[73] Bromm B, Scharein E (1982). Response plasticity of pain evoked reactions in man. Physiol. Behav..

[74] Cowen R, Stasiowska MK, Laycock H, Bantel C (2015). Assessing pain objectively: the use of physiological markers. Anaesthesia.

[75] Rhudy JL, Bartley EJ, Williams AE (2010). Habituation, sensitization, and emotional valence modulation of pain responses. Pain.

[76] Slepian PM (2017). Behavioral inhibition and behavioral activation are related to habituation of nociceptive flexion reflex, but not pain ratings. J. Pain.

[77] France CR, France JL (2002). al’Absi, M., Ring, C. & McIntyre, D. Catastrophizing is related to pain ratings, but not nociceptive flexion reflex threshold. Pain.

[78] Koh CW, Drummond PD (2006). Dissociation between pain and the nociceptive blink reflex during psychological arousal. Clin. Neurophysiol..

[79] García-Larrea L, Charles N, Sindou M, Mauguière F (1993). Flexion reflexes following anterolateral cordotomy in man: dissociation between pain sensation and nociceptive reflex RIII. Pain.

[80] Egger MD (1978). Sensitization and habituation of dorsal horn cells in cats. J. Physiol. (Lond).

[81] Groves PM, Thompson RF (1970). Habituation: a dual-process theory. Psychol. Rev..

[82] Groves PM, De Marco R, Thompson RF (1969). Habituation and sensitization of spinal interneuron activity in acute spinal cat. Brain Res..

[83] Ikeda H (2006). Synaptic amplifier of inflammatory pain in the spinal dorsal horn. Science (80-.)..

[84] Schlereth T, Magerl W, Treede R (2001). Spatial discrimination thresholds for pain and touch in human hairy skin. Pain.

[85] Mouraux A, Iannetti GD (2008). Across-trial averaging of event-related EEG responses and beyond. Magn. Reson. Imaging.

[86] Makeig S, Jung TP, Bell AJ, Ghahremani D, Sejnowski TJ (1997). Blind separation of auditory event-related brain responses into independent components. Proc. Natl. Acad. Sci. USA.

[87] Twisk, J. W. R. *Applied multilevel analysis*. *A practical guide*. (Cambridge University Press, Cambridge, UK, 2005).

[88] Gross J, Schnitzler A, Timmermann L, Ploner M (2007). Gamma oscillations in human primary somatosensory cortex reflect pain perception. PLoS Biol..

[89] Schulz E, Tiemann L, Schuster T, Gross J, Ploner M (2011). Neurophysiological coding of traits and states in the perception of pain. Cereb. Cortex.

[90] Pfurtscheller G, da Silva L (1999). F. H. Event-related EEG/MEG synchronization and desynchronization: basic principles. Clin. Neurophysiol..

[91] Hu L, Xiao P, Zhang ZG, Mouraux A, Iannetti GD (2014). Single-trial time-frequency analysis of electrocortical signals: baseline correction and beyond. Neuroimage.

