# Supplementary material for: Habituation of phase-locked local field potentials and gamma-band oscillations recorded from the human insula
Source: Sci Rep. 2018 May 29;8:8265. doi: 10.1038/s41598-018-26604-0 (PMC5974133; doi:10.1038/s41598-018-26604-0)
Supplement: Supplementary file 1 — Supplemental material [file 41598_2018_26604_MOESM1_ESM.pdf]

Habituation of phase-locked local field potentials and gamma-band oscillations recorded from the human insula

Giulia Liberati PhD*[1], Maxime Algoet[1], Anne Klöcker PhD[1], Susana Ferrao Santos MD[2], Jose Geraldo Ribeiro-Vaz MD[3], Christian Raftopoulos MD[3], André Mouraux MD, PhD[1]

[1]Institute of Neuroscience, Université catholique de Louvain, 1200 Brussels, Belgium.

[2]Department of Neurology, Saint-Luc University Hospital, 1200 Brussels, Belgium.

[3]Department of Neurosurgery, Saint-Luc University Hospital, 1200 Brussels, Belgium.

*Correspondence to: giulia.liberati@uclouvain.be
Avenue Mounier 53, 1200 Brussels, Belgium
Tel: 02 764 54 47

high-intensity stimuli

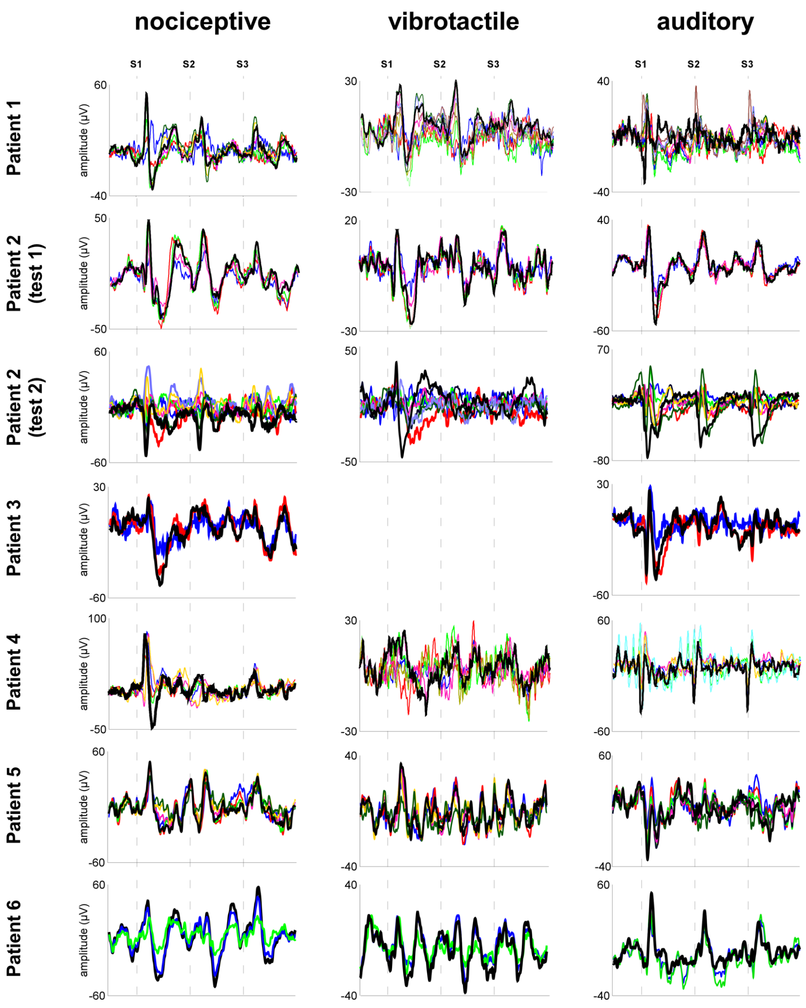

Supplemental material 1 Low-frequency phase-locked local field potentials (LFPs) elicited in the human insula by high-intensity nociceptive, vibrotactile, and auditory "triplets". All types of stimuli elicited low-frequency phase-locked LFPs in the insula, appearing as large biphasic waves. Each waveform corresponds to the averaged low-frequency phase-locked LFP recorded at each individual insular contact. For each participant, the low-frequency phase-locked LFP with the largest amplitude is shown in black. For all three modalities, low-frequency phase-locked LFPs elicited by the first stimuli of the triplets (S1) were greater in amplitude than low frequency phase-locked LFPs elicited by the second (S2) and third (S3) stimuli of the triplets.

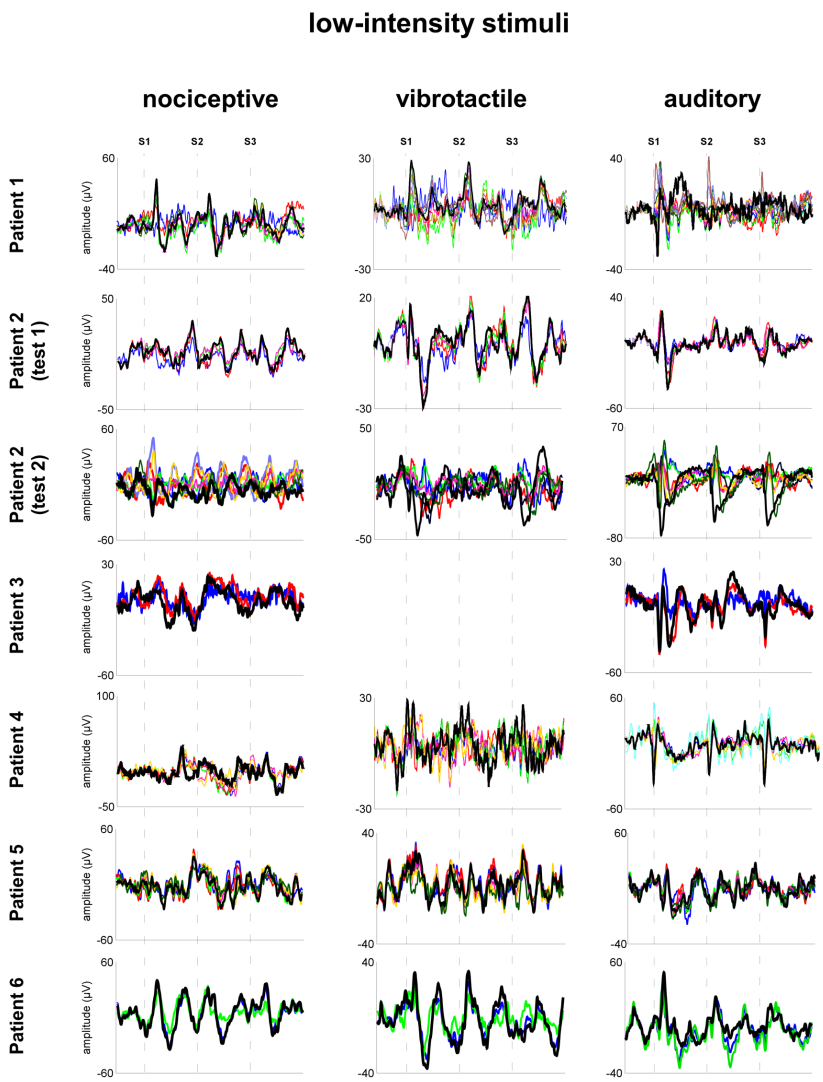

Supplemental material 2 Low-frequency phase-locked local field potentials (LFPs) elicited in the human insula by low-intensity nociceptive, vibrotactile, and auditory "triplets". Each waveform corresponds to the averaged low-frequency phase-locked LFP recorded at each individual insular contact. For each participant, the low-frequency phase-locked LFP with the largest amplitude is shown in black. For the auditory modality, low-frequency phase-locked LFPs elicited by the first stimuli of the triplets (S1) were greater in amplitude than low-frequency phase-locked LFPs elicited by the second (S2) and third (S3) stimuli of the triplets.
